# Supplementary figures and images for: Identification of Flap endonuclease 1 as a potential core gene in hepatocellular carcinoma by integrated bioinformatics analysis
Source: PeerJ. 2019 Sep 6;7:e7619. doi: 10.7717/peerj.7619 (PMC6733258; doi:10.7717/peerj.7619)

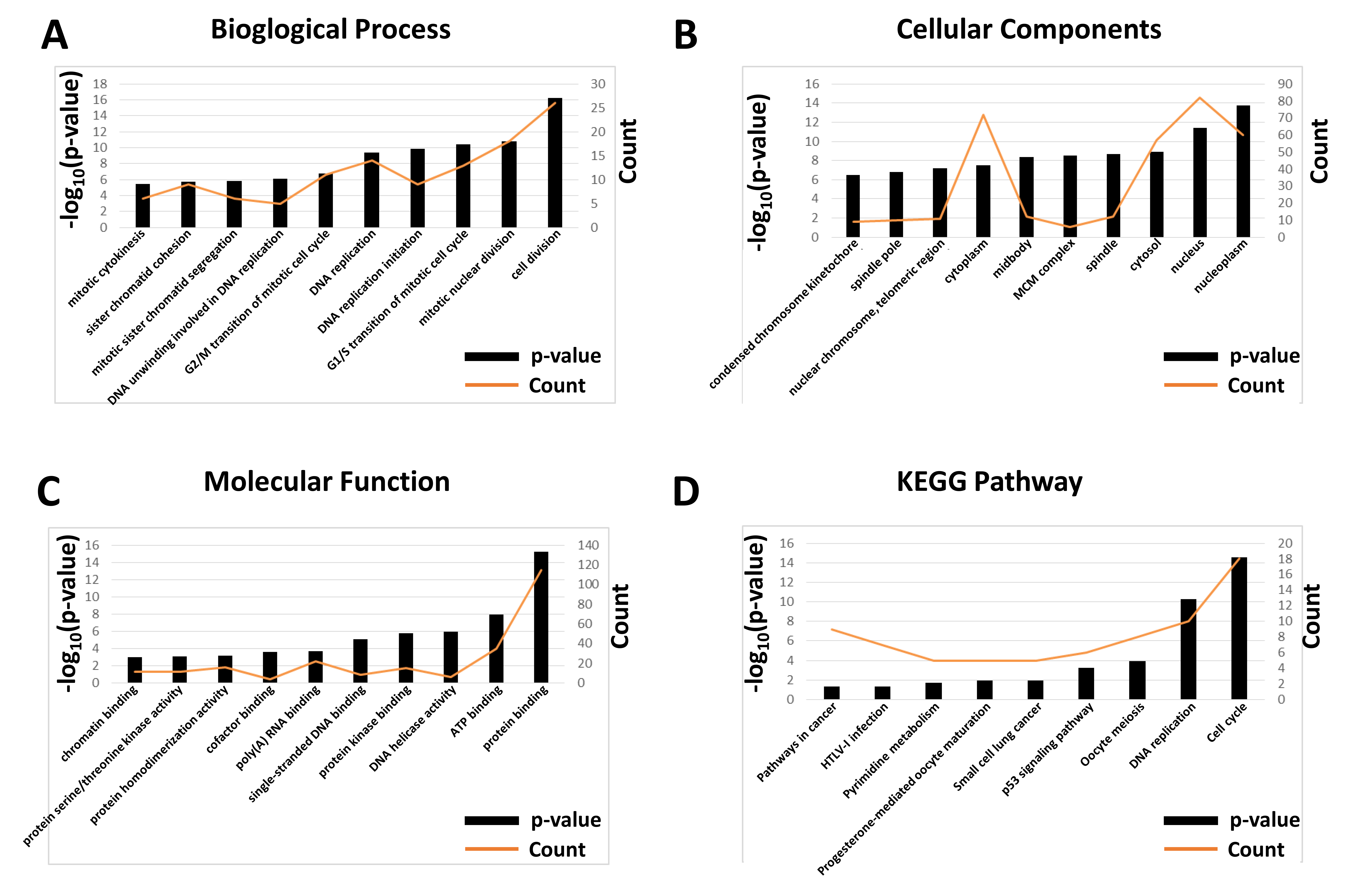

Supplement: Figure S1 — Enriched biological processes (A), cellular components (B), and molecular functions (C). (D) Enriched KEGG pathways among upregulated DEGs. GO terms/KEGG pathways are shown on the x-axis, while y-axes detail x –log10 (P values) and numbers of DEGs. [file peerj-07-7619-s001.png]

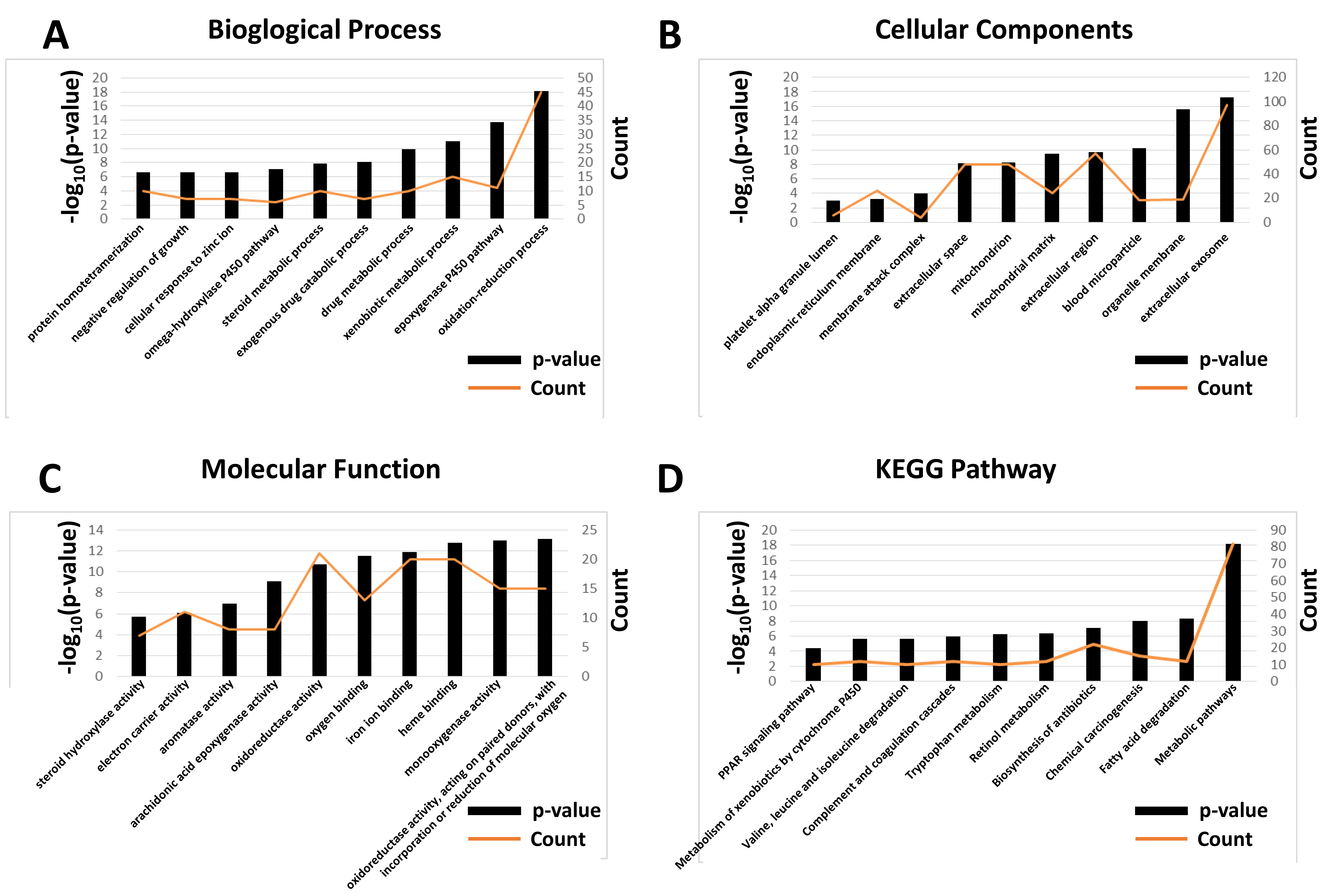

Supplement: Figure S2 — Enriched biological processes (A), cellular components (B), and molecular functions (C). (D) Enriched KEGG pathways among downregulated DEGs. GO terms/KEGG pathways are shown on the x-axis, while y-axes detail x –log10 (P values) and numbers of DEGs. [file peerj-07-7619-s002.png]
