# Supplementary material for: Identification of Flap endonuclease 1 as a potential core gene in hepatocellular carcinoma by integrated bioinformatics analysis
Source: PeerJ. 2019 Sep 6;7:e7619. doi: 10.7717/peerj.7619 (PMC6733258; doi:10.7717/peerj.7619)
Supplement: Table S1 [file peerj-07-7619-s003.docx]

**Table S1** A total of 409 DEGs were identified from four profile datasets, including 142 upregulated and 267 downregulated genes in HCC compared with normal tissues

| DEGs | Gene names |
| --- | --- |
| Upregulated DEGs | GPC3, SPINK1, PEG10, ACSL4, RRM2, TOP2A, ASPM, SQLE, KIAA0101, ROBO1, CCNB1, PTTG1, KPNA2, CDK1, ZWINT, PRC1, NUSAP1, CDKN3, CENPF, UBD, CKS2, GINS1, SULT1C2, LAPTM4B, MDK, AURKA, DTL, GMNN, RACGAP1, RFC4, PLCB1, MCM6, ECT2, SOX4, COL4A1, CENPU, CNIH4, HMMR, ENAH, CDC20, IGF2BP3, SMC4, NCAPG, ILF2, ANXA2, NEK2, FEN1, BIRC5, UBE2C, EZH2, HMGB2, BUB1B, HN1, COL15A1, SNRPD1, ATAD2, KIF20A, SMYD3, MELK, TTK, TPX2, H2AFX, IGF2BP2, PLXNC1, CDKN2, TKT, MCM2, ACLY, PBK, SAC3D1, RRAGD, MCM3, PSPH, ASPH, LRRC1, CCT6A, MAD2L1, PRIM1, RRP15, CKAP2, UBAP2L, PRKDC, DLG5, RFX5, ITGA6, PFDN4, CCNE2, MCM5, CPD, SNRPB, KIF4A, TP53I3, CBX1, BOLA2B, CCNB2, CACYBP, NPM1, ISG20L2, RCN2, GBAP1, NRAS, RAD51AP1, CKAP4, NDC80, DLGAP5, CPSF6, ASNS, GOLPH3L, CCNA2, MSH2, MCM4, IRAK1, HEY1, POLE2, MKI67, MRPL9, STMN1, RRM1, MCM7, SUB1, HMGN4, MMP12, TACC3, FANCI, FOXM1, SERPINI1, KNTC1, PRCC, COL4A2, SLC6A8, HS2ST1, DNAJC6, MEP1A, STK39, NUP155, ALDOA, IGSF3, DTYMK, WHSC1, HJURP, HELLS, VRK1 |
| Downregulated DEGs | TNXB, MPC1, DHODH, GPM6A, KAZN, SLC16A4, TBXA2R, CHRD, SOCS2, ADGRG6, PRG4, TSKU, CYP3A5, CPT2, ABHD6, FGA, SLC23A2, CCL21, NPC1L1, BCKDHB, PON1, SLC28A1, ETS2, HMGCS2, SERPING1, SHBG, AR, CAT, LPAL2, SARDH, FYN, PLPP3, IGFBP4, RUNDC3B, GREM2, TFR2, STARD5, ABCB4, NAAA, PDLIM5, ALDH6A1, RAB17, CA5A, GPD1, CDC14B, CYP4F12, HMGCL, ACADL, CYP4F3, SRD5A1, HPGD, ASL, NPY1R, MPDZ, N4BP2L1, ACOX2, CYP3A43, HGD, PAMR1, AASS, CTSO, MME, DUSP1, CBR4, SULT1A2, PLIN1, CLU, CHST4, LYVE1, SLC27A2, PFKFB1, SLC6A12, PDK4, GADD45B, ACAA2, MS4A6A, SULT1A1, FAM149A, MSRA, FAM13A, GRHPR, ALDH1B1, CFP, MASP2, APCS, PEMT, CDA, ACADSB, ZFP36, HP, ECM2, RIDA, CYP2C9, CETP, HAL, CSAD, ALDH2, PRODH2, PCK2, ACAA1, C1RL, PLG, CDC37L1, HPR, HAAO, HPX, CYP1A1, SYNE1, TKFC, MTHFD1, IGF1, GCGR, RCAN1, ACSL1, MAN1C1, MTTP, GABARAPL3, PCOLCE, ALDOB, GNE, CYP2J2, CYP4F2, GCH1, ECHDC2, OTC, ITIH4, HGF, RNF125, ACACB, ABAT, OLFML3, ETFDH, PTH1R, ST3GAL6, SLC38A4, ALDH1L1, CA2, GCDH, ASPA, CD1D, SEC14L2, AZGP1, CPEB3, GCKR, C1R, NR1I3, GRAMD1C, FAM134B, SORL1, CYP2B6, KDM8, MAT1A, C8B, DCXR, CD14, CIDEB, STAB2, EPHX2, ASS1, ADH6, OLH1B, SLC46A3, STEAP3,ADRA1A, ACSM3, MARCO, CLEC4M, BDH2, LY6E, AZGP1P1, PBLD, IGFALS, FETUB, RCL1, PROZ, CYP2B7P, CYP26A1, HBA2, RND3, PLGLB1, OGDHL, ENO3, ACSM5, MFAP3L, AKR7A3, CTH, FTCD, KMO, F11, TDO2, CLEC1B, ALDH8A1, CRHBP, CYP2C19, CDHR2, ZGPAT, FXYD1, ECM1, TAT, CD5L, CXCL12, NR1I2, BHMT, GSTZ1, C6, FBP1, BBOX1, ANXA10, KLKB1, MT2A, CFHR4, CYP4A11, ETNPPL, C8A, ADH1B, SRPX, SRD5A2, MT1HL1, DCN, SLC27A5, FCN2, F9, GHR, CXCL2, KCNN2, LPA, ID1, EGR1, GLYAT, ESR1, CYP3A4, MT1M, VIPR1, RDH16, AFM, HSD11B1, PCK1, ABCA8, DNASE1L3, GNMT, LCAT, HGFAC, MT1X, SLC10A1, GBA3, ADH1C, MT1H, SDS, HPD, MT1E, FCN3, GYS2, AKR1D1, GLS2, CYP39A1, CXCL14, MT1G, HAO2, MT1F, CYP2A6, SLCO1B3, APOF, C9, NAT2, SLC22A1, CYP1A2, HAMP |
