# Supplementary material for: Identification of Flap endonuclease 1 as a potential core gene in hepatocellular carcinoma by integrated bioinformatics analysis
Source: PeerJ. 2019 Sep 6;7:e7619. doi: 10.7717/peerj.7619 (PMC6733258; doi:10.7717/peerj.7619)
Supplement: Table S2 [file peerj-07-7619-s004.docx]

**Table S2** Gene function analysis of clustering module 1

| Term | Count | PValue |
| --- | --- | --- |
| Bioglogical Process |  |  |
| GO:0051301~cell division | 23 | 5.50E-22 |
| GO:0007067~mitotic nuclear division | 16 | 8.73E-15 |
| GO:0006260~DNA replication | 14 | 9.56E-15 |
| GO:0000082~G1/S transition of mitotic cell cycle | 12 | 7.77E-14 |
| GO:0006270~DNA replication initiation | 9 | 1.85E-13 |
| GO:0000086~G2/M transition of mitotic cell cycle | 10 | 1.49E-09 |
| GO:0007062~sister chromatid cohesion | 9 | 3.42E-09 |
| GO:0031145~anaphase-promoting complex-dependent catabolic process | 8 | 1.38E-08 |
| GO:0000070~mitotic sister chromatid segregation | 6 | 2.69E-08 |
| GO:0006268~DNA unwinding involved in DNA replication | 5 | 3.26E-08 |
| Cellular Components |  |  |
| GO:0005654~nucleoplasm | 41 | 7.08E-19 |
| GO:0005634~nucleus | 49 | 3.34E-15 |
| GO:0030496~midbody | 11 | 1.47E-11 |
| GO:0042555~MCM complex | 6 | 4.43E-11 |
| GO:0000777~condensed chromosome kinetochore | 9 | 4.65E-10 |
| GO:0000922~spindle pole | 9 | 2.86E-09 |
| GO:0005819~spindle | 9 | 6.56E-09 |
| GO:0005737~cytoplasm | 39 | 4.63E-08 |
| GO:0005829~cytosol | 30 | 1.49E-07 |
| GO:0000784~nuclear chromosome, telomeric region | 8 | 2.53E-07 |
| Molecular Function |  |  |
| GO:0005515~protein binding | 57 | 3.38E-12 |
| GO:0005524~ATP binding | 24 | 4.86E-10 |
| GO:0003678~DNA helicase activity | 6 | 1.93E-08 |
| GO:0019901~protein kinase binding | 10 | 6.65E-06 |
| GO:0003682~chromatin binding | 9 | 6.89E-05 |
| GO:0003697~single-stranded DNA binding | 5 | 3.32E-04 |
| GO:0003677~DNA binding | 16 | 5.94E-04 |
| GO:0008017~microtubule binding | 6 | 8.53E-04 |
| GO:0004674~protein serine/threonine kinase activity | 7 | 0.0021318 |
| GO:0042826~histone deacetylase binding | 4 | 0.0057110 |
| KEGG Pathway |  |  |
| hsa04110:Cell cycle | 16 | 1.80E-19 |
| hsa03030:DNA replication | 10 | 9.33E-15 |
| hsa04114:Oocyte meiosis | 8 | 2.74E-07 |
| hsa04115:p53 signaling pathway | 5 | 1.63E-04 |
| hsa04914:Progesterone-mediated oocyte maturation | 5 | 4.45E-04 |
| hsa00240:Pyrimidine metabolism | 4 | 0.00851012 |
| hsa05166:HTLV-I infection | 5 | 0.02085069 |
| hsa00230:Purine metabolism | 4 | 0.03695496 |
