# Supplementary material for: Identification of Flap endonuclease 1 as a potential core gene in hepatocellular carcinoma by integrated bioinformatics analysis
Source: PeerJ. 2019 Sep 6;7:e7619. doi: 10.7717/peerj.7619 (PMC6733258; doi:10.7717/peerj.7619)
Supplement: Table S3 [file peerj-07-7619-s005.docx]

**Table S3** Gene function analysis of clustering module 2

| Term | Count | PValue |
| --- | --- | --- |
| Bioglogical Process |  |  |
| GO:0031639~plasminogen activation | 3 | 3.46E-05 |
| GO:0007597~blood coagulation, intrinsic pathway | 3 | 1.46E-04 |
| GO:0019835~cytolysis | 3 | 2.00E-04 |
| GO:0030449~regulation of complement activation | 3 | 4.13E-04 |
| GO:0007596~blood coagulation | 4 | 7.86E-04 |
| GO:0043420~anthranilate metabolic process | 2 | 0.00303426 |
| GO:0019805~quinolinate biosynthetic process | 2 | 0.00303426 |
| GO:0006956~complement activation | 3 | 0.00343093 |
| GO:0034354~'de novo' NAD biosynthetic process from tryptophan | 2 | 0.00404376 |
| GO:0051919~positive regulation of fibrinolysis | 2 | 0.00404376 |
| Cellular Components |  |  |
| GO:0070062~extracellular exosome | 14 | 3.52E-08 |
| GO:0005576~extracellular region | 10 | 4.05E-06 |
| GO:0005615~extracellular space | 9 | 1.16E-05 |
| GO:0005579~membrane attack complex | 3 | 1.72E-05 |
| GO:1903561~extracellular vesicle | 2 | 0.0456515 |
| Molecular Function |  |  |
| GO:0004252~serine-type endopeptidase activity | 5 | 1.04E-04 |
| GO:0005496~steroid binding | 2 | 0.02685775 |
| GO:0008134~transcription factor binding | 3 | 0.03247997 |
| GO:0004879~RNA polymerase II transcription factor activity, ligand-activated sequence-specific DNA binding | 2 | 0.03565842 |
| GO:0019899~enzyme binding | 3 | 0.04341188 |
| KEGG Pathway |  |  |
| hsa04610:Complement and coagulation cascades | 7 | 1.33E-09 |
| hsa05020:Prion diseases | 3 | 0.00178763 |
| hsa05322:Systemic lupus erythematosus | 3 | 0.02552765 |
