# Supplementary material for: Identification of Flap endonuclease 1 as a potential core gene in hepatocellular carcinoma by integrated bioinformatics analysis
Source: PeerJ. 2019 Sep 6;7:e7619. doi: 10.7717/peerj.7619 (PMC6733258; doi:10.7717/peerj.7619)
Supplement: Table S4 [file peerj-07-7619-s006.docx]

**Table S4** Gene function analysis of clustering module 3

| Term | Count | PValue |
| --- | --- | --- |
| Bioglogical Process |  |  |
| GO:0008202~steroid metabolic process | 9 | 6.59E-16 |
| GO:0006805~xenobiotic metabolic process | 10 | 7.68E-16 |
| GO:0042738~exogenous drug catabolic process | 7 | 3.97E-15 |
| GO:0019373~epoxygenase P450 pathway | 7 | 7.94E-14 |
| GO:0017144~drug metabolic process | 7 | 1.26E-12 |
| GO:0097267~omega-hydroxylase P450 pathway | 5 | 4.02E-10 |
| GO:0042737~drug catabolic process | 4 | 5.12E-08 |
| GO:0016098~monoterpenoid metabolic process | 4 | 5.12E-08 |
| GO:0070989~oxidative demethylation | 4 | 5.60E-07 |
| GO:0055114~oxidation-reduction process | 9 | 1.02E-06 |
| Cellular Components |  |  |
| GO:0031090~organelle membrane | 10 | 1.03E-15 |
| GO:0005789~endoplasmic reticulum membrane | 10 | 7.82E-07 |
| GO:0034364~high-density lipoprotein particle | 4 | 3.04E-06 |
| GO:0043231~intracellular membrane-bounded organelle | 8 | 5.34E-06 |
| GO:0034366~spherical high-density lipoprotein particle | 3 | 4.63E-05 |
| GO:0072562~blood microparticle | 4 | 0.00101243 |
| GO:0005576~extracellular region | 8 | 0.00373723 |
| GO:0034362~low-density lipoprotein particle | 2 | 0.01828669 |
| GO:0005615~extracellular space | 6 | 0.02856822 |
| Molecular Function |  |  |
| GO:0019825~oxygen binding | 9 | 1.36E-15 |
| GO:0020037~heme binding | 10 | 1.38E-13 |
| GO:0005506~iron ion binding | 10 | 3.79E-13 |
| GO:0016705~oxidoreductase activity, acting on paired donors, with incorporation or reduction of molecular oxygen | 8 | 1.13E-12 |
| GO:0004497~monooxygenase activity | 8 | 1.29E-12 |
| GO:0016712~oxidoreductase activity, acting on paired donors, with incorporation or reduction of molecular oxygen, reduced flavin or flavoprotein as one donor, and incorporation of one atom of oxygen | 6 | 1.11E-11 |
| GO:0008395~steroid hydroxylase activity | 6 | 2.40E-10 |
| GO:0008392~arachidonic acid epoxygenase activity | 5 | 4.24E-09 |
| GO:0070330~aromatase activity | 5 | 5.39E-08 |
| GO:0016491~oxidoreductase activity | 6 | 7.86E-06 |
| KEGG Pathway |  |  |
| hsa05204:Chemical carcinogenesis | 9 | 6.64E-13 |
| hsa00830:Retinol metabolism | 8 | 1.41E-11 |
| hsa00982:Drug metabolism - cytochrome P450 | 7 | 2.10E-09 |
| hsa00980:Metabolism of xenobiotics by cytochrome P450 | 7 | 3.54E-09 |
| hsa01100:Metabolic pathways | 11 | 1.48E-05 |
| hsa00591:Linoleic acid metabolism | 4 | 2.38E-05 |
| hsa00232:Caffeine metabolism | 3 | 3.83E-05 |
| hsa00140:Steroid hormone biosynthesis | 4 | 1.94E-04 |
| hsa00983:Drug metabolism - other enzymes | 3 | 0.00378268 |
| hsa00590:Arachidonic acid metabolism | 3 | 0.00657268 |
