# Supplementary material for: Identification of Flap endonuclease 1 as a potential core gene in hepatocellular carcinoma by integrated bioinformatics analysis
Source: PeerJ. 2019 Sep 6;7:e7619. doi: 10.7717/peerj.7619 (PMC6733258; doi:10.7717/peerj.7619)
Supplement: Table S5 [file peerj-07-7619-s007.docx]

**Table S5** The core genes in clustering module 1were screened by 12 algorithms of cytoHubba plug-in

| **node name** | **MCC** | **DMNC** | **MNC** | **Degree** | **EPC** | **BottleNeck** | **EcCentricity** | **Closeness** | **Radiality** | **Betweenness** | **Stress** | **ClusteringCoefficient** |
| --- | --- | --- | --- | --- | --- | --- | --- | --- | --- | --- | --- | --- |
| GINS1 | 9.22E+13 | 1.51098 | 44 | 44 | 8.08 | 1 | 0.5 | 52.5 | 1.77049 | 0.2296 | 12 | 0.99366 |
| CKAP2 | 9.22E+13 | 1.50849 | 45 | 45 | 8.444 | 1 | 0.5 | 53 | 1.78689 | 0.59359 | 30 | 0.98485 |
| HJURP | 9.22E+13 | 1.5448 | 49 | 49 | 7.912 | 1 | 0.5 | 55 | 1.85246 | 0.91026 | 44 | 0.98129 |
| EZH2 | 9.22E+13 | 1.54346 | 49 | 49 | 8.805 | 1 | 0.5 | 55 | 1.85246 | 0.91233 | 46 | 0.98044 |
| RRM1 | 9.22E+13 | 1.55079 | 51 | 51 | 8.827 | 1 | 0.5 | 56 | 1.88525 | 1.43114 | 70 | 0.97255 |
| GMNN | 9.22E+13 | 1.54829 | 51 | 51 | 9.629 | 1 | 0.5 | 56 | 1.88525 | 1.46563 | 74 | 0.97098 |
| FOXM1 | 9.22E+13 | 1.56947 | 54 | 54 | 9.203 | 1 | 0.5 | 57.5 | 1.93443 | 1.94226 | 96 | 0.96646 |
| MCM5 | 9.22E+13 | 1.55004 | 52 | 52 | 9.168 | 1 | 0.5 | 56.5 | 1.90164 | 1.85891 | 90 | 0.96606 |
| PRIM1 | 9.22E+13 | 1.56833 | 54 | 54 | 9.717 | 1 | 0.5 | 57.5 | 1.93443 | 2.03121 | 98 | 0.96576 |
| CCNE2 | 9.22E+13 | 1.54762 | 52 | 52 | 9.336 | 1 | 0.5 | 56.5 | 1.90164 | 1.90355 | 94 | 0.96456 |
| NEK2 | 9.22E+13 | 1.5638 | 54 | 54 | 9.424 | 1 | 0.5 | 57.5 | 1.93443 | 2.20035 | 106 | 0.96296 |
| KPNA2 | 9.22E+13 | 1.56039 | 54 | 54 | 9.636 | 1 | 0.5 | 57.5 | 1.93443 | 2.44361 | 112 | 0.96087 |
| CDKN3 | 9.22E+13 | 1.58588 | 57 | 57 | 9.87 | 1 | 0.5 | 59 | 1.98361 | 2.6856 | 128 | 0.9599 |
| ATAD2 | 9.22E+13 | 1.54868 | 53 | 53 | 8.87 | 1 | 0.5 | 57 | 1.91803 | 2.3643 | 112 | 0.95936 |
| MCM6 | 9.22E+13 | 1.58381 | 57 | 57 | 10.103 | 1 | 0.5 | 59 | 1.98361 | 2.77456 | 132 | 0.95865 |
| MCM3 | 9.22E+13 | 1.57459 | 56 | 56 | 9.436 | 1 | 0.5 | 58.5 | 1.96721 | 2.69926 | 128 | 0.95844 |
| CKS2 | 9.22E+13 | 1.57352 | 56 | 56 | 10.143 | 1 | 0.5 | 58.5 | 1.96721 | 2.74808 | 130 | 0.95779 |
| PTTG1 | 9.22E+13 | 1.58174 | 57 | 57 | 10.026 | 1 | 0.5 | 59 | 1.98361 | 2.89044 | 136 | 0.95739 |
| POLE2 | 9.22E+13 | 1.57245 | 56 | 56 | 10.255 | 1 | 0.5 | 58.5 | 1.96721 | 2.81075 | 132 | 0.95714 |
| CENPF | 9.22E+13 | 1.58993 | 58 | 58 | 9.782 | 1 | 0.5 | 59.5 | 2 | 2.96685 | 142 | 0.95705 |
| FANCI | 9.22E+13 | 1.58893 | 58 | 58 | 10.424 | 1 | 0.5 | 59.5 | 2 | 3.02086 | 144 | 0.95644 |
| MKI67 | 9.22E+13 | 1.58792 | 58 | 58 | 10.192 | 1 | 0.5 | 59.5 | 2 | 3.06041 | 146 | 0.95584 |
| MCM7 | 9.22E+13 | 1.57864 | 57 | 57 | 9.643 | 1 | 0.5 | 59 | 1.98361 | 2.99527 | 142 | 0.95551 |
| HMMR | 9.22E+13 | 1.5776 | 57 | 57 | 9.859 | 1 | 0.5 | 59 | 1.98361 | 3.03976 | 144 | 0.95489 |
| CENPU | 9.22E+13 | 1.57657 | 57 | 57 | 9.968 | 1 | 0.5 | 59 | 1.98361 | 3.09377 | 146 | 0.95426 |
| FEN1 | 9.22E+13 | 1.58491 | 58 | 58 | 10.045 | 1 | 0.5 | 59.5 | 2 | 3.21976 | 152 | 0.95402 |
| ECT2 | 9.22E+13 | 1.5745 | 57 | 57 | 9.82 | 1 | 0.5 | 59 | 1.98361 | 3.17973 | 150 | 0.95301 |
| HELLS | 9.22E+13 | 1.5445 | 54 | 54 | 9.111 | 1 | 0.5 | 57.5 | 1.93443 | 3.05388 | 140 | 0.95108 |
| MCM2 | 9.22E+13 | 1.58832 | 59 | 59 | 10.031 | 1 | 0.5 | 60 | 2.01639 | 3.58369 | 168 | 0.95091 |
| KNTC1 | 9.22E+13 | 1.57787 | 58 | 58 | 9.562 | 1 | 0.5 | 59.5 | 2 | 3.62788 | 166 | 0.94979 |
| NDC80 | 9.22E+13 | 1.58637 | 59 | 59 | 9.738 | 1 | 0.5 | 60 | 2.01639 | 3.72762 | 172 | 0.94974 |
| DTL | 9.22E+13 | 1.59292 | 60 | 60 | 10.152 | 1 | 0.5 | 60.5 | 2.03279 | 3.8797 | 182 | 0.94859 |
| MCM4 | 9.22E+13 | 1.59292 | 60 | 60 | 9.76 | 1 | 0.5 | 60.5 | 2.03279 | 3.8797 | 182 | 0.94859 |
| SMC4 | 9.22E+13 | 1.58149 | 59 | 59 | 9.7 | 1 | 0.5 | 60 | 2.01639 | 3.96617 | 182 | 0.94681 |
| RFC4 | 9.22E+13 | 1.58912 | 60 | 60 | 10.156 | 1 | 0.5 | 60.5 | 2.03279 | 4.10516 | 190 | 0.94633 |
| KIF4A | 9.22E+13 | 1.58723 | 60 | 60 | 10.161 | 1 | 0.5 | 60.5 | 2.03279 | 4.22142 | 194 | 0.9452 |
| KIF20A | 9.22E+13 | 1.58723 | 60 | 60 | 9.713 | 1 | 0.5 | 60.5 | 2.03279 | 4.22142 | 194 | 0.9452 |
| KIAA0101 | 9.22E+13 | 1.58628 | 60 | 60 | 10.3 | 1 | 0.5 | 60.5 | 2.03279 | 4.27424 | 196 | 0.94463 |
| DLGAP5 | 9.22E+13 | 1.58628 | 60 | 60 | 9.799 | 1 | 0.5 | 60.5 | 2.03279 | 4.27022 | 196 | 0.94463 |
| BIRC5 | 9.22E+13 | 1.58533 | 60 | 60 | 10.176 | 1 | 0.5 | 60.5 | 2.03279 | 4.32613 | 198 | 0.94407 |
| CDC20 | 9.22E+13 | 1.58533 | 60 | 60 | 10.118 | 1 | 0.5 | 60.5 | 2.03279 | 4.32613 | 198 | 0.94407 |
| PRC1 | 9.22E+13 | 1.58438 | 60 | 60 | 9.988 | 2 | 0.5 | 60.5 | 2.03279 | 4.38158 | 200 | 0.9435 |
| AURKA | 9.22E+13 | 1.58438 | 60 | 60 | 9.986 | 1 | 0.5 | 60.5 | 2.03279 | 4.38158 | 200 | 0.9435 |
| RAD51AP1 | 9.22E+13 | 1.58438 | 60 | 60 | 9.877 | 1 | 0.5 | 60.5 | 2.03279 | 4.37075 | 200 | 0.9435 |
| UBE2C | 9.22E+13 | 1.58937 | 61 | 61 | 10.278 | 2 | 1 | 61 | 2.04918 | 4.66283 | 214 | 0.94153 |
| NUSAP1 | 9.22E+13 | 1.58937 | 61 | 61 | 10.637 | 1 | 1 | 61 | 2.04918 | 4.66283 | 214 | 0.94153 |
| PBK | 9.22E+13 | 1.58937 | 61 | 61 | 10.558 | 1 | 1 | 61 | 2.04918 | 4.66283 | 214 | 0.94153 |
| RRM2 | 9.22E+13 | 1.58937 | 61 | 61 | 10.47 | 1 | 1 | 61 | 2.04918 | 4.66283 | 214 | 0.94153 |
| CCNA2 | 9.22E+13 | 1.58937 | 61 | 61 | 10.416 | 1 | 1 | 61 | 2.04918 | 4.66283 | 214 | 0.94153 |
| NCAPG | 9.22E+13 | 1.58937 | 61 | 61 | 10.384 | 1 | 1 | 61 | 2.04918 | 4.66283 | 214 | 0.94153 |
| CDK1 | 9.22E+13 | 1.58937 | 61 | 61 | 10.363 | 1 | 1 | 61 | 2.04918 | 4.66283 | 214 | 0.94153 |
| RACGAP1 | 9.22E+13 | 1.58937 | 61 | 61 | 10.292 | 1 | 1 | 61 | 2.04918 | 4.66283 | 214 | 0.94153 |
| TTK | 9.22E+13 | 1.58937 | 61 | 61 | 10.255 | 1 | 1 | 61 | 2.04918 | 4.66283 | 214 | 0.94153 |
| BUB1B | 9.22E+13 | 1.58937 | 61 | 61 | 10.24 | 1 | 1 | 61 | 2.04918 | 4.66283 | 214 | 0.94153 |
| TPX2 | 9.22E+13 | 1.58937 | 61 | 61 | 10.147 | 1 | 1 | 61 | 2.04918 | 4.66283 | 214 | 0.94153 |
| ZWINT | 9.22E+13 | 1.58937 | 61 | 61 | 10.125 | 1 | 1 | 61 | 2.04918 | 4.66283 | 214 | 0.94153 |
| ASPM | 9.22E+13 | 1.58937 | 61 | 61 | 9.991 | 1 | 1 | 61 | 2.04918 | 4.66283 | 214 | 0.94153 |
| TOP2A | 9.22E+13 | 1.58937 | 61 | 61 | 9.986 | 1 | 1 | 61 | 2.04918 | 4.66283 | 214 | 0.94153 |
| MAD2L1 | 9.22E+13 | 1.58937 | 61 | 61 | 9.949 | 1 | 1 | 61 | 2.04918 | 4.66283 | 214 | 0.94153 |
| CCNB2 | 9.22E+13 | 1.58937 | 61 | 61 | 9.89 | 1 | 1 | 61 | 2.04918 | 4.66283 | 214 | 0.94153 |
| CCNB1 | 9.22E+13 | 1.58937 | 61 | 61 | 9.835 | 1 | 1 | 61 | 2.04918 | 4.66283 | 214 | 0.94153 |
| MELK | 9.22E+13 | 1.58937 | 61 | 61 | 9.755 | 1 | 1 | 61 | 2.04918 | 4.66283 | 214 | 0.94153 |
