# Supplementary material for: Identification of Flap endonuclease 1 as a potential core gene in hepatocellular carcinoma by integrated bioinformatics analysis
Source: PeerJ. 2019 Sep 6;7:e7619. doi: 10.7717/peerj.7619 (PMC6733258; doi:10.7717/peerj.7619)
Supplement: Table S6 [file peerj-07-7619-s008.docx]

**Table S6** 185 Hub genes were screened and identified by CytoHubba plugin

| Filter condition | Gene names |
| --- | --- |
| degree≥10 | CDK1, CCNA2, CCNB1, MAD2L1, RFC4, TOP2A, AURKA, CCNB2, CDC20, RRM2, KPNA2, TTK, PBK, BIRC5, UBE2C, NDC80, TPX2, NCAPG, BUB1B, MELK, ASPM, NUSAP1, FOXM1, SMC4, RACGAP1, MCM4, MCM2, MKI67, FEN1, MCM7, DLGAP5, KIF20A, ZWINT, KIAA0101, FANCI, RAD51AP1, CDKN3, PRC1, KIF4A, MCM6, EZH2, DTL, HMMR, PTTG1, MCM3, KNTC1, CENPF, CENPU, CKS2, ECT2, RRM1, HELLS, POLE2, MCM5, ATAD2, PRIM1, NEK2, CCNE2, GMNN, HJURP, CKAP2, H2AFX, TACC3, CAT, GINS1, HMGB2, FTCD, FGA, ESR1, MSH2, AR, HP, HGD, CLU, CYP3A4, IGF1, FETUB, PCK1, CYP2B6, TAT, VRK1, PLG, HAO2, F9, ACLY, STMN1, C8A, PCK2, HMGCS2, CYP2C9, NUP155, CYP1A1, LPA, ACAA1, HPX, CTH, DTYMK, KLKB1, HGF, ASS1, C6, CPT2, CYP1A2, ALDOA, ALDH8A1, PON1, C9, ACSL1, ACADL, HMGCL, CYP2C19, ALDOB, ACAA2, HAAO, KMO, SERPING1, ITIH4, CYP4A11, HGFAC, NPM1, NR1I2, BHMT, PRKDC, CFP, ACOX2, F11, FBP1, LCAT, SULT1A1, ACSM3, CYP3A5, CYP26A1, CYP2A6, CXCL12, C1R, C8B, GYS2, SLC10A1, GNMT, EGR1, SDS, NR1I3, EPHX2, ALDH2, ETFDH, PEMT, ENO3, DCN, MAT1A, ACACB, ASL, SULT1C2, CDA, ALDH1B1, GCDH, SLC27A2, PSPH, MTHFD1, TKT, HPD, OTC, AFM, PDK4, PROZ, SQLE, SLCO1B3, ILF2, CYP4F2, ALDH6A1, ACADSB, NAT2, CCT6A, HAL, GPD1, SORL1, SARDH, WHSC1, NRAS, HPR, APCS, GRHPR, HSD11B1, CYP2J2, CETP, SNRPD1 |
